# Supplementary material for: Challenging the highstand-dormant paradigm for land-detached submarine canyons
Source: Nat Commun. 2022 Jun 15;13:3448. doi: 10.1038/s41467-022-31114-9 (PMC9199327; doi:10.1038/s41467-022-31114-9)
Supplement: Supplementary file 1 — Supplementary Information [file 41467_2022_31114_MOESM1_ESM.pdf]

Supplementary figures and tables for "Challenging the highstand-dormant paradigm for land-detached submarine canyons" by Heijnen et al.

This file presents supplementary material including the timing of earthquakes during the monitoring period (Table S1), photographs of the sediment trap and evidence of tangled fishing gear (Figure S1), grain size distribution of samples from the sediment trap (Figure S2), a global overview of similar canyon systems (Figure S3), and a plan view visualisation of bottom-disturbing fishing effort around the Whittard Canyon head (Figure S4).

Table S1: Magnitude 2 and larger earthquakes reported within 1000 km of the head of the eastern branch of Whittard Canyon during the monitoring period.

Date	Magnitude	Latitude	Longitude	Distance from Whittard Canyon head
19/04/2020	2.6	39.969°N	8.518°W	986 km
19/02/2020	3.7	48.376°N	4.447°W	419 km
23/01/2020	2.8	54.577°N	1.335°W	884 km
05/12/2019	3.4	51.012°N	3.079°W	562 km
26/08/2019	2.3	53.798°N	3.180°W	737 km

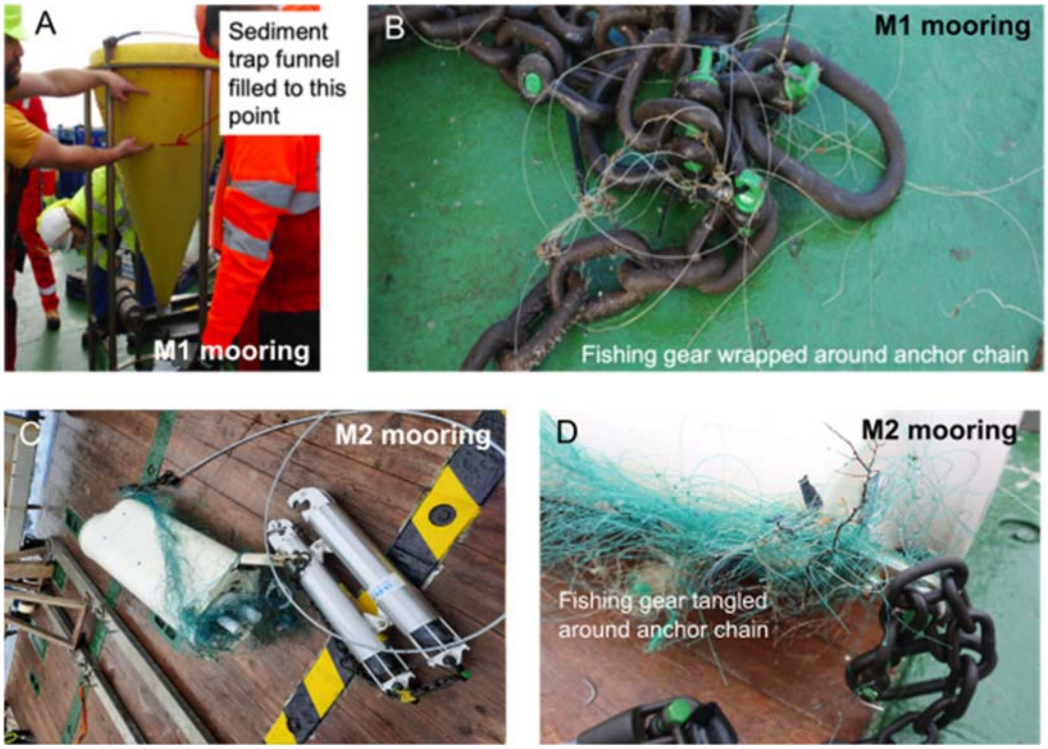

Figure S1: Photographs taken at sea during recovery of moorings. Recovery of Mooring M1 found (A) sediment fill in the mooring funnel above the carousel that holds the sampling bottles (left), and (B) the anchor chain that was wrapped in discarded fishing gear. Photographs taken at sea during recovery of Mooring M2 show (C) release links

and snagged fishing gear and (D) detail on fishing gear tangled around the anchor chain.

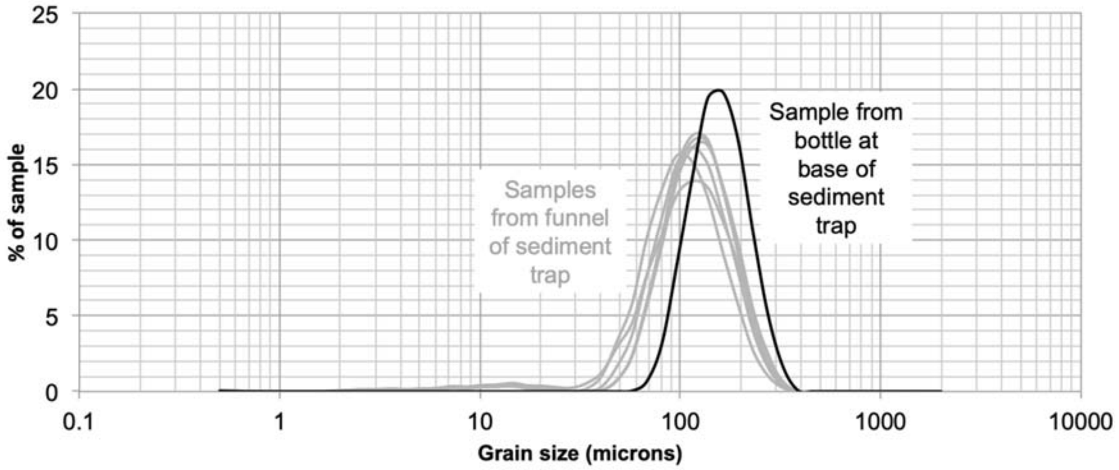

Figure S2: Grain size distribution of sediment sampled from a sediment trap located 10 m above seafloor on Mooring M1. Grain size analysis was performed on samples taken from the sediment trap funnel, and the sampling bottle at the base. This sediment accumulation occurred within the first 18 days of deployment and is interpreted to be associated with flow 1.

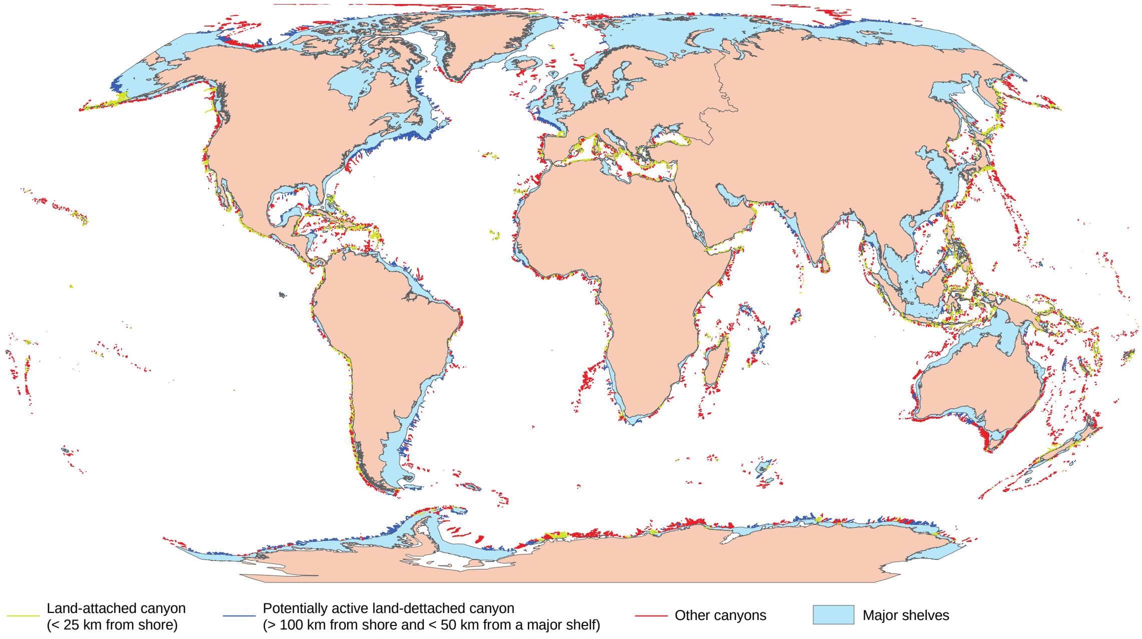

Figure S3: Distribution of the N=9477 submarine canyons worldwide as mapped by Harris and Whiteway[1]. We define land-attached canyons as those within 25 km of shore and account for 22% (N=2104). Land-detached canyons, which lie at least 100 km from shore account for 12% (N=1162). Note we exclude canyons on the Antarctic shelf from this analysis as they may be fed in different ways (i.e. by cascading dense cold water, rather than sediment flows). The remaining canyons ("other canyons") account for 66%

(N=6211). This figure uses supplementary data from ‘Global distribution of large submarine canyons: Geomorphic differences between active and passive continental margins’, Volume 285, Issues 1-4, Pages 69-86, Harris, P.T. and Whiteway, T. (2011), with permission from Elsevier under Licence Number 5310751500640.

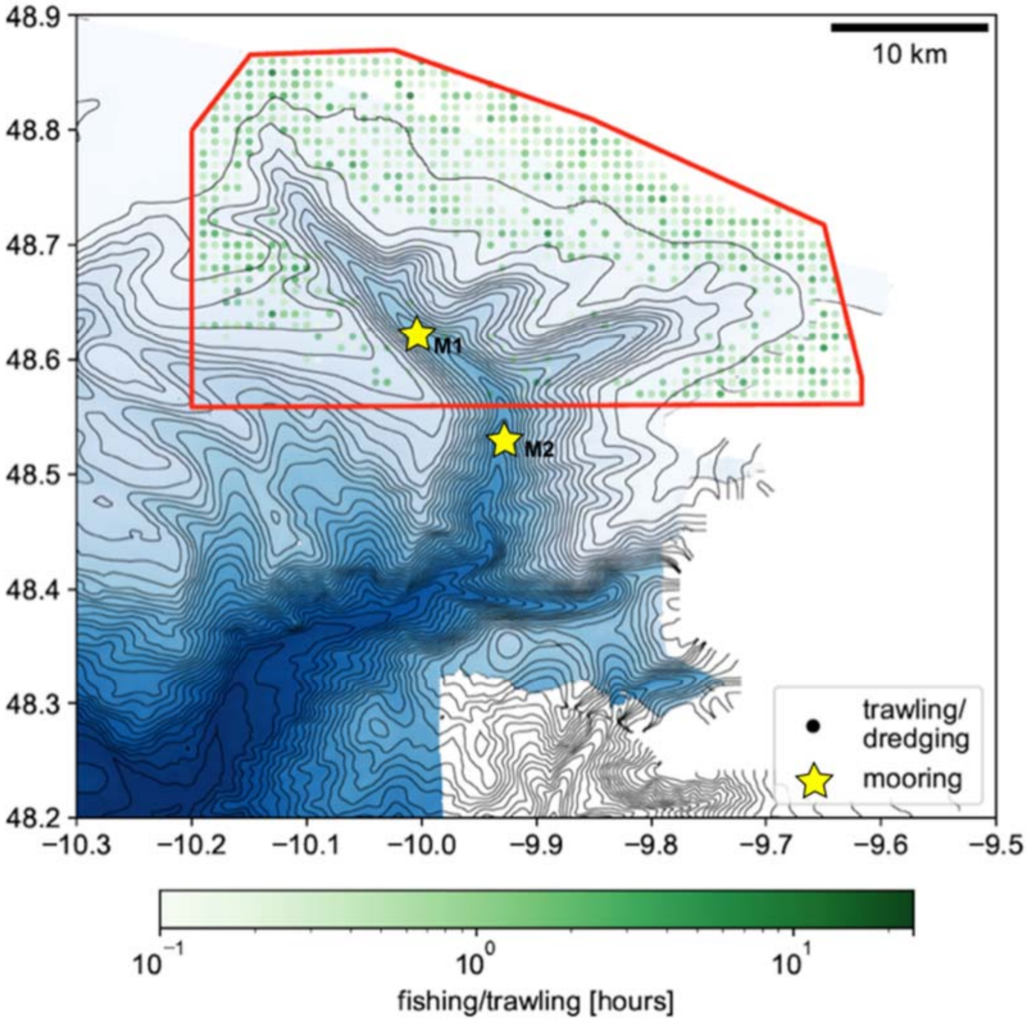

Figure S4: Intensity of bottom dredging and trawling as recorded by Global Fishing Watch (https://globalfishingwatch.org/). Green filled circles show total hours during which fishing activities that disturb the seafloor were occurring within the monitoring period. Mooring M1 shown as a red triangle.
